# Enhancing Time-Frequency Analysis with Zero-Mean Preprocessing

**DOI:** 10.3390/s22072477

**Published:** 2022-03-23

**Authors:** Sunghyun Jin, Philip Johansson, HeeSeok Kim, Seokhie Hong

**Affiliations:** 1 School of Cyber Security, Korea University, Seoul 02841, Korea; sunghyunjin@korea.ac.kr; 2 Center for Information Security Technologies, Institute of Cyber Security and Privacy, Korea University, Seoul 02841, Korea; philipjohansson2021@gmail.com; 3 Department of Cyber Security, College of Science and Technology, Korea University, Sejong 30019, Korea; 80khs@korea.ac.kr

**Keywords:** second-order side-channel analysis, time-frequency analysis, Fourier transform, masking, hiding, desynchronization

## Abstract

Side-channel analysis is a critical threat to cryptosystems on the Internet of Things and in relation to embedded devices, and appropriate side-channel countermeasure must be required for physical security. A combined countermeasure approach employing first-order masking and desynchronization simultaneously is a general and cost-efficient approach to counteracting side-channel analysis. With the development of side-channel countermeasures, there are plenty of advanced attacks introduced to defeat such countermeasures. At CARDIS 2013, Belgarric et al. first proposed time-frequency analysis, a promising attack regarding the complexity of computation and memory compared to other attacks, such as conventional second-order side-channel analysis after synchronization. Nevertheless, their time-frequency analysis seems to have lower performance than expected against some datasets protected by combined countermeasures. It is therefore required to study the factors that affect the performance of time-frequency analysis. In this paper, we investigate Belgarric et al.’s time-frequency analysis and conduct a mathematical analysis in regard to the preprocessing of frequency information for second-order side-channel analysis. Based on this analysis, we claim that zero-mean preprocessing enhances the performance of time-frequency analysis. We verify that our analysis is valid through experimental results from two datasets, which are different types of first-order masked Advanced Encryption Standard (AES) software implementations. The experimental results show that time-frequency analysis with zero-mean preprocessing seems to have an enhanced or complementary performance compared to the analysis without preprocessing.

## 1. Introduction

Cryptosystems play an important role in modern electronic systems, providing security mechanisms such as data encryption and authentication. Cryptographic algorithms are designed to be mathematically secure, and then they are implemented and operated on semiconductors. However, when the cryptographic algorithms operate, semiconductors leak unintentional information such as execution time [1], power consumption [2], electromagnetic radiation [3,4], sound [5], and temperature [6] related to internal instruction and data. Side-channel analysis (SCA) is cryptanalysis using the side-channel leakages. Because the leakages include direct/indirect information on secret key, and SCA has the nature of a divide-and-conquer strategy, vastly decreasing its computation cost, SCA is a practical threat, more efficient than other methods of algorithmic cryptanalysis such as differential/linear-like statistical cryptanalysis. Furthermore, the rapid growth of edge devices, such as embedded devices and the Internet of Things (IoT), identifies SCA as an actively ongoing research field for physical security, even though it was first introduced by Kocher in the late 1990s [7].

SCA can be classified into profiling and non-profiling attacks [8,9]. Although profiling attacks, such as template attack [10,11] and deep learning-based side-channel analysis, [12,13,14] are more powerful than non-profiling attacks, it has the constraint of requiring a profiling device, which is the same physical form as a target device. Due to this constraint, non-profiling attacks are more appropriate in the black box scenario. Representative non-profiling attacks are differential power analysis (DPA) [2] and correlation power analysis (CPA) [15]. Both are statistical attacks that exploit the relation between leakage information and a hypothesis of the internal value calculated from the partial plaintext and a partial secret key. Because the valid statistic appears only when the hypothesis is calculated with a correct key guess, an attacker can guess the secret key correctly through these statistical attacks. The only difference between the two types of attacks is using different statistics as distinguishers: the difference of mean and the correlation coefficient, respectively. Because both attacks are partitioning power attacks [16], we only consider CPA in the rest of this paper. Since CPA easily breaks naïve implementation on cryptographic algorithms, it must be protected by appropriate side-channel countermeasures.

A masking scheme is a representative countermeasure approach for defending against CPA [17,18,19,20,21]. It removes the relationship between side-channel leakages and data by concealing all internal values, including plaintext, ciphertext, and a secret key, using uniform random values. In a masking scheme, the masking order is a major security parameter and is determined according to the maximum number of masks for any value. In general, sound dth-order masking protects all below dth-order analysis techniques. However, since the masking order increases the cost of implementation exponentially, it can be impractical to use a higher-order masking scheme unconditionally without appropriate threat modeling, or even with proper threat modeling. Another countermeasure approach is thus required.

The hiding scheme is another approach for counteracting SCA. It decreases the signal-to-noise ratio (SNR) through creating constant side-channel leakage [22,23,24,25] or causing desynchronization such as random delay [26,27], jitter, or shuffling [18,28]. Although the hiding scheme does not provide theoretically perfect security against SCA, it is popularly used because it sufficiently increases attack complexity and is cheaper than a masking scheme, i.e., it provides practical security. However, since solely employing a hiding method cannot provide full security, an attacker could break a cryptosystem protected by such a scheme if the attacker can improve the SNR of traces by collecting sufficient traces or using appropriate preprocessing methods, such as alignment [8,29,30], noise reduction [31,32,33,34,35,36,37,38,39,40], and even the latest deep learning-based side-channel preprocessing [41]. For those reasons, another approach combining masking and hiding schemes concurrently is commonly used. This approach provides practical security and a reasonable implementation cost. For example, the Advanced Encryption Standard (AES) [42] is commonly protected by the combined countermeasures of first-order masking and hiding in real-world scenarios [43].

With the development of side-channel countermeasures, studies on advanced SCA such as higher-order SCA [44,45], zero-value/offset SCA [21,46], and differential deep learning-based analysis [47] against cryptosystems protected by the countermeasures have also continued. For defeating masking, preprocessing by combining leakages on shares of target value, such as absolute difference and product combining, must be performed. Because combining all shares on an intermediate value reveals the first-order leakage, CPA can be accomplished easily after the combining preprocessing. This CPA including the preprocessing, which has a mechanism for combining shares, is called higher-order CPA. For higher-order CPA, the attacker must carefully and accurately choose points of interest (PoI), a collection of time indexes of trace corresponding to shares. However, because this PoI selection is not a trivial task in non-profiled attack scenarios, some ranges are chosen to include the accurate time sample on shares, i.e., ground truth [48]. On the other hand, because preprocessing increases the length of processed traces exponentially, depending on the number of PoI, the size of the range is limited regarding computational and memory cost. Furthermore, in the case of the masking and desynchronization-based hiding combined countermeasure, neutralizing the desynchronization, such as static trace alignment and dynamic time wrapping-based alignment, must be conducted before the higher-order CPA. If any step of the attack against the combined countermeasure fails, the attack ultimately fails.

As an alternative approach for higher-order CPA against the combined countermeasure, at CARDIS 2013, Belgarric et al. proposed time-frequency analysis (TFA), which is an improved variant of FFT-2DPA [46] for CPA, [49]. TFA is a CPA on transformed traces, which are postprocessed through auto-correlation (auto-corr), cross-correlation (x-corr), or absolute value after being preprocessed through DFT or a discrete Hartley transform (DHT). Compared to the aforementioned higher-order CPA, the frequency-based transform is a role of desynchronization [50], and postprocessing is a role of mixing shares [49]. In the perspective of attacker ability, TFA attacker is less required to have the ability to synchronize traces compared to the higher-order CPA attacker because the Fourier transform is time-shift invariant. Furthermore, although there is a tradeoff regarding SNR, TFA attacker requires less capability to conduct PoI selection because the Fourier transform is a linear operation, and rough PoI range selection creates correct first-order leakage after the postprocessing. Fourier transform maintains the trace length, and it is a major strength in the complexity of computation and memory. Thus, TFA is a promising tool for side-channel attackers and evaluators. Nevertheless, when TFA is used, it occasionally fails to analyze some datasets protected by the combined countermeasure approach. We thus investigate what causes the difference between success and failure when TFA is utilized. Similar to the latest work [51] that studies how to overcome poor performance due to the ghost peak in DPA using mathematical analysis and by employing normalized variance-based pre/post-processing, we study the reason for TFA’s poor performance through mathematical analysis.

In this paper, we first mathematically investigate Belgarric et al.’s TFA against first-order masking and hiding countermeasures and how TFA can remove the masking and hiding countermeasures. We explore the mathematical analysis on equations for the postprocessing of TFA. Based on this analysis, we claim that applying zero-mean preprocessing is necessary before frequency transform to enhance second-order SCA against cryptosystems protected by the combined countermeasure approach. We propose using standardization and min-max normalization methods as zero-mean preprocessing. We then validate our claim through experimental results on two datasets of different first-order masked AES software implementations. The experimental results show that TFA with zero-mean preprocessing seems to demonstrate either an enhanced or a complementary performance compared to TFA without the zero-mean preprocessing.

## 2. Preliminaries

We use the following notations: Let the capital letter X denote random variables, and the lowercase x denote their realizations. The j-th entry of a vector x is defined as $x\left[j\right]$. Let $T\in R^{D\times 𝟙}$ denote side-channel traces of length D. The targeted sensitive variable is $Z=f\left(P,K\right)$ where f denotes a part of a cryptographic primitive, P denotes a public variable such as (partial) plaintext or ciphertext, and K denotes a (partial) secret key.

### 2.1. Correlation Power Analysis

The CPA exploits the correlation between a device’s power consumption and the hypothesis of the data generated during computation. The correlation coefficient ρ between actual power consumption and the values of the predicted leakage model (ex. Hamming weight) of hypothesis is calculated as below:

(1) $$\rho \left(T,H\right)=\frac{Cov\left(T,H\right)}{\sqrt{Var\left(T\right)\cdot Var\left(H\right)}},$$

where variable T represents a set of real power consumption values, H is a set of predicted Hamming weight values, $Cov\left(X,Y\right)$ is the covariance of X and Y, and $Var\left(X\right)$ is the variance of X. If the key guess is correct, then the hypothesis is calculated correctly, and the correlation coefficient is higher than other cases on incorrect key guesses.

### 2.2. Masking Countermeasure

CPA is an attack to find a secret key by analyzing the correlation between power consumption traces and the intermediate values of a cryptographic algorithm. Such an attack can be counteracted by removing the relationship between the two data groups. The masking countermeasure is a mainstream and unique means to provide provable security against CPA.

Masking is implemented by using uniform random values, i.e., the mask value, to conceal the intermediate. For every execution of the crypto algorithm, new mask values are generated, and these random values are employed to hide all cryptographically calculated intermediate values. Algorithm 1 is a first-order masking AES-128 example using Boolean masking [18]. It is noted that the crypto algorithm is operated correctly, although the intermediate values are concealed by the masking values.
**Algorithm 1:** First-order masked AES-128**Input**: Plaintext $PT\left[16\right]$, Master key $MK\left[16\right]$, AES S-box $S\left[256\right]$ **Output**: Ciphertext $CT\left[16\right]$ **Initialization** 1: Choose masking values $r_{in}$, $r_{out}$, $m_{1}$, $m_{2}$, $m_{3}$, $m_{4}$ uniformly at random from ${𝕫}_{256}$ 2: Initialize MS 3: **for**
i∈0 up to 255 do 4: $MS\left[i⊕r_{in}\right]=S\left[i\right]⊕r_{out}$ 5: **end for** 6: $m_{1}^{\prime },\;m_{2}^{\prime },\;m_{3}^{\prime },\;m_{4}^{\prime }\leftarrow MixColumns\left(m_{1},m_{2},m_{3},m_{4}\right)$ 7: $state\leftarrow Remask\left(PT,\;0,\;m_{1}^{\prime },\;m_{2}^{\prime },\;m_{3}^{\prime },\;m_{4}^{\prime }\right)$ 8: $rk\leftarrow KeySchedule\left(MK,\;r_{in},\;r_{out},\;m_{1}^{\prime },\;m_{2}^{\prime },\;m_{3}^{\prime },\;m_{4}^{\prime }\right)$ **Encryption** 9: $state\leftarrow AddRoundKey\left(state,\;rk\right)$ 10: **for** i∈1 up to 9 do 11:   $state\leftarrow SubBytes\left(state,\;MS\right)$ 12:   $state\leftarrow ShiftRows\left(state\right)$ 13:   $state\leftarrow Remask\left(state,\;r_{out},\;m_{1},\;m_{2},\;m_{3},\;m_{4}\right)$ 14:   $state\leftarrow MixColumns\left(state\right)$ 15:   $state\leftarrow AddRoundKey\left(state,\;rk\right)$ 16: **end for** 17: $state\leftarrow SubBytes\left(state,\;MS\right)$ 18: $state\leftarrow ShiftRows\left(state\right)$ 19: $CT\leftarrow AddRoundKey\left(state,\;rk\right)$ 20: **Return**
CT


### 2.3. Hiding Countermeasure

The hiding scheme is another approach for counteracting SCA by decreasing the signal-to-noise ratio (SNR). The hiding scheme reduces the relation between traces and data, which are the side-channel leakages and intermediate value during cryptosystem operations. Hiding is a traditional countermeasure to SCA, and it sufficiently increases attack complexity while being cheaper than a masking scheme. However, if the attacker can use more traces or employ advanced preprocessing methodologies, such as alignment, noise reduction, or even the latest deep learning-based side-channel preprocessing, hiding countermeasures can be neutralized. Therefore, hiding is not currently used alone, but is widely used as a secondary means together with other countermeasures.

### 2.4. Second-Order Correlation Power Analysis

Second-order CPA (SO-CPA) is an attack method capable of analyzing first-order masking countermeasures. SO-CPA is equal to the original CPA, except for a preprocess which combines the two shares related on a single sensitive intermediate value. In this paper, we adhere to the assumptions and notations of Prouff et al. for SO-CPA [45]. We also concentrate on the product combining method to explore TFA from among the variable preprocessing methods for SO-CPA.

Let a sensitive value $x\in Z_{m}$ be separated into two shares $x_{0},\;x_{1}$ using a uniform random value $r\in Z_{m}$ as follows:

(2) $$x_{0}=x⊕r,x_{1}=r,$$

The leakage of each share is defined as follows:

(3) $$L_{i}=L\left(x_{i}\right)\;=H\left(x_{i}\right)\;+\;\delta_{i}+b_{i},$$

where $\delta_{i}$ is a constant term of the leakage, $b_{i}$ is an instance of a Gaussian random variable $b_{i}∼N\left(0,\sigma \right),$ and H is a hypothesis, e.g., Hamming weight or Hamming distance. Next, product combining, a representative preprocess for SO-CPA, can be applied to traces as follows:

(4) $$L^{\prime }=C_{prod1}\left(L_{0},L_{1}\right)\;=L_{0}\cdot L_{1},$$

or

(5) $$L^{\prime \prime }=C_{prod2}\left(L_{0},L_{1}\right)\;=\;\left(L_{0}-E\left[L_{0}\right]\right)\cdot \left(L_{1}-E\left[L_{1}\right]\right),$$

We denote the two versions of product combining as prod1 and prod2. After such product combining, CPA might succeed against preprocessed traces $L^{\prime }$ or $L^{\prime \prime }$ targeting the sensitive intermediate value x.

### 2.5. Time-Frequency Analysis for Second-Order Side-Channel Analysis

TFA is one method for solving the hiding countermeasure by applying frequency transform through DFT (Definition 1) and DHT (discrete Hartley transform) and the masking countermeasure by applying postprocessing such as auto-correlation, cross-correlation, or absolute value for combining the shares’ leakage points. TFA can thus analyze the two countermeasures, masking and hiding, at once.

**Definition** **1.** *Given a sequence* $X\in R^{n}$ *, the discrete Fourier transform of* X *is calculated as follows:*

(6) $$DFT\left(X\right)\left[f\right]=\frac{1}{\sqrt{n}}\sum_{t=0}^{n-1}X\left[t\right]\cdot \exp\left(-i\cdot \frac{2\pi ft}{n}\right)$$

where 0≤f<n and i∈C is the imaginary unit, i.e., $i^{2}=-1$.

Belgarric et al. propose five analysis methods that combine the DFT or DHT with auto-corr or x-corr, and exploit other correlations using max-corr as a heuristic method. Belgarric et al. finally asserted that future studies should investigate TFA’s potential for higher-order SCA because postprocessing can be naturally adjusted for it. In the next section, we investigate how Belgarric et al.’s TFA work for defeating masking and hiding combined countermeasures.

## 3. Mathematical Analysis on Time-Frequency Analysis with Zero-Mean Preprocessing

In this section, we explicitly investigate Belgarric et al.’s TFA and explore detailed mathematical formulas for TFA postprocessing. Then, based on this mathematical analysis, we claim that applying zero-mean preprocessing followed by TFA must be required to improve the performance of the attack.

For the simplicity of representing our mathematical analysis, we have made some assumptions. First, we assume that two sensitivity leakages are included in a single time interval; thus, we only explore the absolute value postprocessing method, since other cases can be easily analyzed in the same manner. Second, we focus on DFT rather than DHT because DHT can also be easily analyzed in the same manner as our first assumption.

### 3.1. Mathematical Analysis

By Euler’s formula, $DFT\left(X\right)\left[f\right]$ in Equation (6) can be represented as follows:

(7) $$DFT\left(X\right)\left[f\right]=\frac{1}{\sqrt{n}}\left[\left(\sum_{t=0}^{n-1}X\left[t\right]\cdot \cos\left(\frac{2\pi ft}{n}\right)\right)-i\cdot \left(\sum_{t=0}^{n-1}X\left[t\right]\cdot \sin\left(\frac{2\pi ft}{n}\right)\right)\right],$$

Since $DFT\left(X\right)$ is a linear combination of X, the two leakage parts on $L_{i}$ are not yet mixed multiplicatively in $DFT\left(X\right)$ for SO-SCA. Thus, a step that mixes leakage parts on two shares multiplicatively is required. For such a mixing step, the absolute value $\left|DFT\left(X\right)\left[f\right]\right|$ is computed as follows:

(8) $$\left|DFT\left(X\right)\left[f\right]\right|=\frac{1}{\sqrt{n}}\sqrt{{\left(\sum_{t=0}^{n-1}X\left[t\right]\cdot \cos\left(\frac{2\pi ft}{n}\right)\right)}^{2}+{\left(\sum_{t=0}^{n-1}X\left[t\right]\cdot \sin\left(\frac{2\pi ft}{n}\right)\right)}^{2}},$$

For simplicity, we only consider index t=0, 1 representing the time samples of $L_{0}$, $L_{1},$ and index t=2,  a time sample irrelevant to sensitivity value, respectively, i.e., two samples of each share and an independent sample. We skip the constant term $1/\sqrt{\left(n\right)}$. Finally, the absolute value can be simplified as follows:

(9) $$\left|DFT\left(X\right)\left[f\right]\right|=\sqrt{\begin{array}{c} \sum_{0\leq t_{1}\neq t_{2}\leq 2}X\left[t_{1}\right]\cdot X\left[t_{2}\right]\cdot \left(\begin{array}{c} \cos\left(\frac{2\pi ft_{1}}{n}\right)\cos\left(\frac{2\pi ft_{2}}{n}\right)\\ +\sin\left(\frac{2\pi ft_{1}}{n}\right)\sin\left(\frac{2\pi ft_{2}}{n}\right) \end{array}\right)\\ +\sum_{t=0}^{2}X^{2}\left[t\right] \end{array}},$$

From Equation (9), we can observe the term of prod1 shown in Equation (4). However, the constant part δ of the leakage model in Equation (3) is not zero, in many cases of the SCA domain. In these cases, prod2 is, in general, superior to prod1 for SO-CPA. Since applying zero-mean preprocessing makes prod1 terms into prod2 terms, applying zero-mean preprocessing will improve the performance of TFA for SO-CPA. We next introduce two candidates for the zero-mean preprocessing.

### 3.2. Zero-Mean Preprocessing

To improve the performance of TFA, we applied zero-mean preprocessing to the traces before converting to the frequency domain. We suggest the following two well-known methods for zero-mean preprocessing.

**Definition** **2.** *(Min-max Normalization) Given* $\left\{X_{j}\in R^{n}\;|\;\;j=0,\;1,\;2,\ldots ,N-1\right\}$ *, the min-max normalization is calculated as follows:*

(10) $${X_{j}}^{\prime }\left[t\right]=\frac{X_{j}\left[t\right]-\min({\left\{X_{j}\left[t\right]\right\}}_{j})}{\max({\left\{X_{j}\left[t\right]\right\}}_{j})-\min({\left\{X_{j}\left[t\right]\right\}}_{j})},$$

**Definition** **3.** *(Standardization) Given* $\left\{X_{j}\in R^{n}\;|\;j=0,1,2,\ldots ,N-1\right\}$ *, standardization is calculated as follows:*

(11) $${X_{j}}^{\prime }\left[t\right]=\frac{\left(X_{j}\left[t\right]-\mu \left({\left\{X_{j}\left[t\right]\right\}}_{j}\right)\right)}{\sigma \left({\left\{X_{j}\left[t\right]\right\}}_{j}\right)},$$

First, standardization performs zero-mean preprocessing, followed by producing a unit variance. Because the unit variance processing does not affect the CPA performance, we can use standardization as zero-mean preprocessing for enhancing TFA. We chose standardization because it is included in many signal processing libraries. Second, although min-max normalization cannot create an exact zero-mean, we expect it to approximate the mean to zero, especially in desynchronized traces, and thus to enhance TFA. It is also included in many signal processing libraries. The next section shows that the experimental results validate our claim.

## 4. Experimental Results

In this section, we describe the experiments we conducted and verify our claim by analyzing the performance changes of TFA whether zero-mean preprocessing is applied or not. We consider three different methodologies, which are the same except for their preprocessing parts. They are denoted as follows in the rest of this paper:

- **TFA** represents Belgarric et al.’s time-frequency analysis without any zero-mean preprocessing.
- **TFAwPS** represents a time-frequency analysis with standardization.
- **TFAwPN** represents a time-frequency analysis with min-max normalization.

We applied each methodology to two datasets that were protected by different first-order masking and a random delay. We repeated each methodology 50 times against each dataset to estimate the guessing entropy per the varying number of traces.

In this section, we show only three methodologies (first-order CPA in the time domain, TFA, and TFAwPS) because we prioritized observing the degree to which zero-mean preprocessing changes performance. Please refer to Appendix A to compare the experimental results on TFAwPS and TFAwPN.

### 4.1. Introduction to the AES-M Datasets

AES-M is a dataset of collected power consumption traces for the simplest form of first-order masked AES with 8-bit software implementation [18]. We acquired 60,000 power traces of AES operation, as shown in Algorithm 1, operating on a CW308T-STM32F target board [52] at 7.37 MHz using a CW1173 ChipWhisperer-Lite [53] with a sampling rate of 29.54 MHz. Note that the power traces acquired from the ChipWhisperer have a high SNR. We then attempted a first-order CPA and confirmed that there was no first-order leakage.

To compare the performance of each methodology in more challenging situations, we simulate desynchronization by only a random shift. In short, we experimented on three cases of AES-M datasets, such as ASCAD dataset with [43]: synchronized traces and two kinds of desynchronization-simulated traces, randomly shifted within 50 and 100 points, respectively. We denote each case as follows: AES-M-sync, AES-M-desync50, and AES-M-desync100. In all the cases, we chose the same two time intervals corresponding to lines 4 and 12 in Algorithm 1 for the points of interest.

### 4.2. Experimental Results on AES-M-Sync

**Figure 1:**
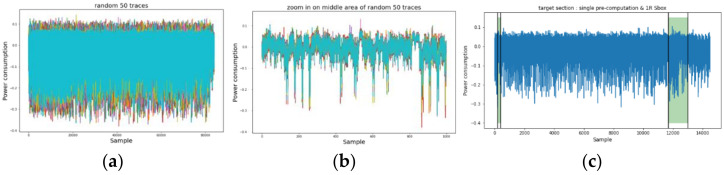
The power consumption traces for the original AES-M-sync dataset in which all traces are synchronized. (**a**) 50 random traces during AES encryptions; (**b**) 50 random traces zoomed on the middle area. (**c**) Single trace zoomed on the front portion, including precomputation, key schedule, and one round with two green boxes. The green boxes represent time intervals for target operations corresponding to lines 4 and 12, respectively, in Algorithm 1.

**Figure 2:**
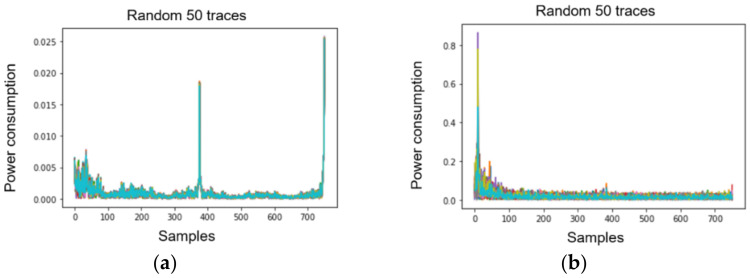
The result of AES-M-sync waveforms transformed by (**a**) TFA; (**b**) TFAwPS.

We first investigated the power traces of AES-M-sync, as shown in Figure 1, and then selected the target time intervals, as shown in Figure 1c. We then transformed the concatenation of the two time intervals for each trace by TFA and TFAwPS, as shown in Figure 2a,b.

Unlike a first-order CPA with raw traces, we succeeded at guessing the correct key using CPA against waveforms that were transformed by both TFA and TFAwPS. This result implies that a transformation based on FFT and absolute value is valid for analyzing first-order masking countermeasures.

**Figure 3:**
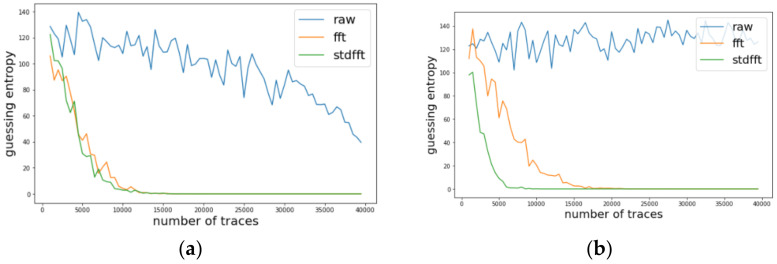
(**a**) The best guessing entropy of TFA results at the 14th byte. (**b**) The best guessing entropy of TFAwPS results at the 15th byte.

**Figure 4:**
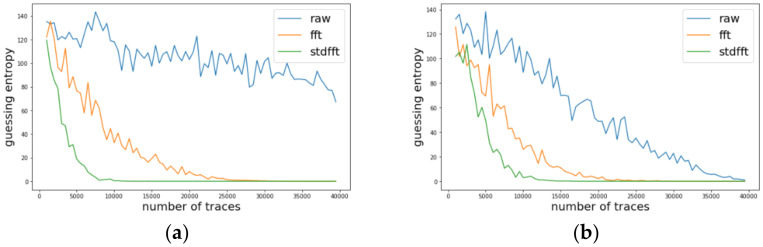
(**a**) The worst guessing entropy of TFA results at 3rd byte. (**b**) Worst guessing entropy of TFAwPS result at 10th byte.

We investigated the change of guessing entropy per varying the number of traces in three cases using basic CPA with raw traces, TFA, and TFAwPS. Figure 3a,b presents the best cases of TFA and TFAwPS, respectively, while Figure 4b presents the worst cases for TFA and TFAwPS, respectively. These results show that TFAwPS performed better than TFA and CPA. Even in the best TFA case, TFAwPS and TFA demonstrate a similar performance; the TFAwPS is equal to or better than TFA. Table 1 summarizes the average performances of TFA and TFAwPS, showing that TFAwPS outperforms TFA.

### 4.3. Experimental Results on AES-M-Desync50

**Figure 5:**
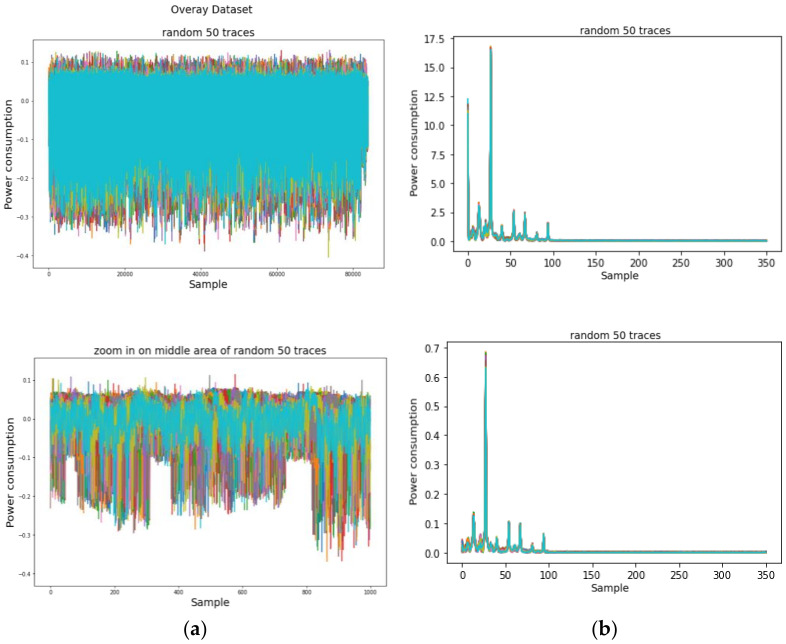
(**a**) The power consumption traces for AES-M-desync50. (**b-up**) The transformed waveforms by TFA. (**b-down**) The transformed waveforms by TFAwPS.

We conducted experiments on AES-M-desync50. Figure 5a presents the traces for AES-M-desync50. As in the previous section, we transformed the concatenation of the two time intervals for each trace using TFA and TFAwPS. As shown in Figure 5b, each transformation by TFA and TFAwPS generates a very similar waveform because the transformation is based on the FFT.

**Figure 6:**
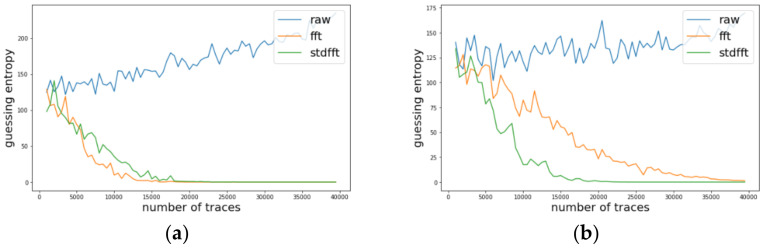
(**a**) The best guessing entropy of TFA results at 12th byte. (**b**) The best guessing entropy of TFAwPS results at 2nd byte.

**Figure 7:**
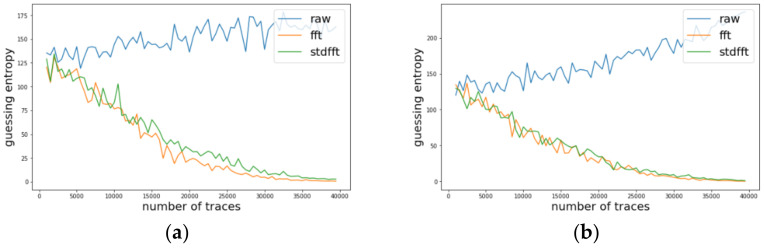
(**a**) the worst guessing entropy of TFA results at 6th byte. (**b**) The worst guessing entropy of TFAwPS results at 9th byte.

Likewise, we investigated the change of guessing entropy per varying the number of traces in three cases investigating a basic CPA with raw traces, TFA, and TFAwPS. Figure 6a,b presents the best cases for TFA and TFAwPS, respectively, while Figure 7a,b presents the worst cases for TFA and TFAwPS, respectively. In the best case, TFAwPS has a better output than TFA and in the other cases, even the worst case, TFAwPS shows a similar performance against AES-M-desync50. Table 2 presents that TFAwPS found one more key than TFA did.

However, concerning the results on all bytes, which are provided in Table A1 in Appendix A, TFA and TFAwPS have a mutual complementary performance regarding each found key byte. We thus conclude that both analyses must be performed together in this dataset case.

### 4.4. Experimental Results on AES-M-Desync100

**Figure 8:**
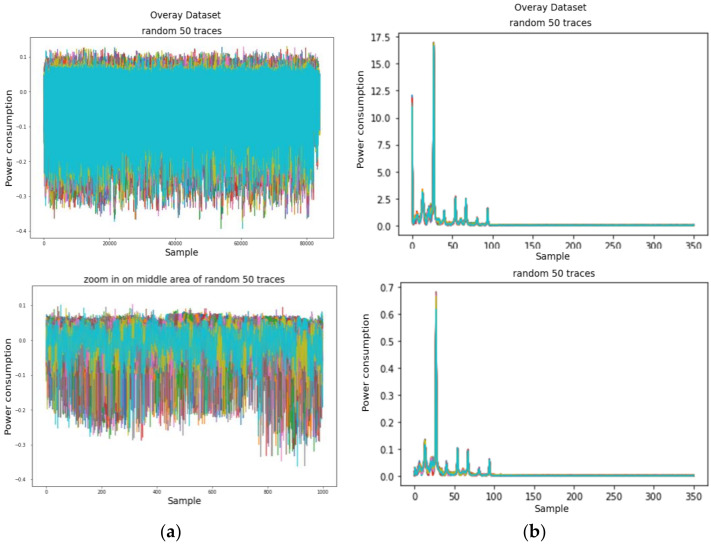
(**a**) The power consumption traces for AES-M-desync100. (**b-up**) The transformed waveforms by TFA. (**b-down**) The transformed waveforms by TFAwPS.

We also experimented on AES-M-desync100. Figure 8a presents the traces for AES-M-desync100. As in the previous section, we transformed the concatenation of the two time intervals for each trace using TFA and TFAwPS. As shown in Figure 8b, each transformation by TFA and TFAwPS also generates a very similar waveform because the transformation is based on the FFT.

**Figure 9:**
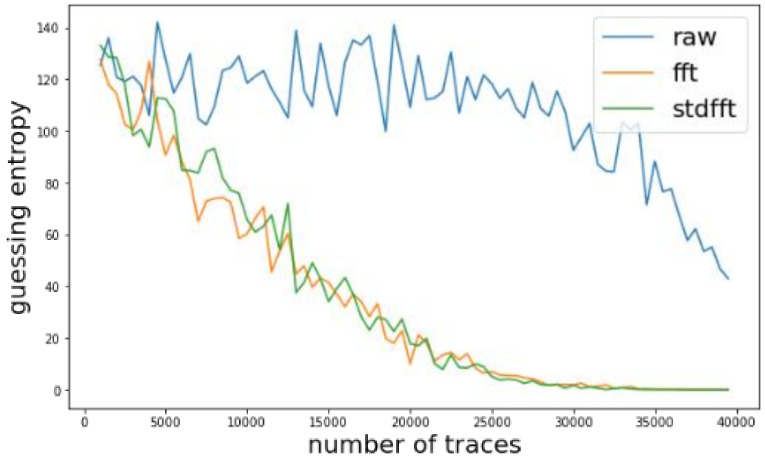
The best guessing entropy of TFA and TFAwPS result at the 13th byte.

**Figure 10:**
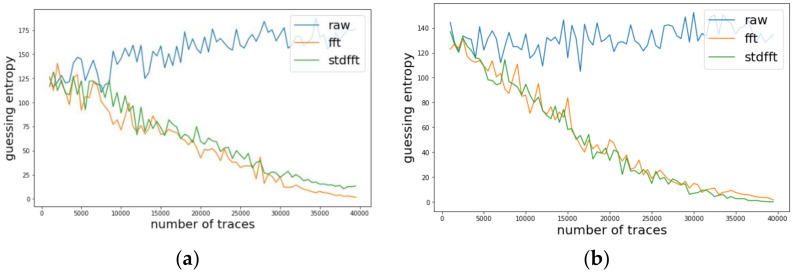
(**a**) The worst guessing entropy of TFA results at 6th byte. (**b**) The worst guessing entropy of TFAwPS result at the 11th byte.

We investigated the change of guessing entropy per varying the number of traces on three cases using a basic CPA with raw traces, TFA, and TFAwPS. Figure 9 present the best cases for TFA and TFAwPS, while Figure 10a,b presents the worst cases for TFA and TFAwPS, respectively. From the best and worst results, we recognize that TFA and TFAwPS achieve similar performances. Due to the use of fixed time intervals and severe random delay, TFA and TFAwPS succeeded at finding only four keys (See Table 3).

This poor performance is related to the efficiency of the analysis, and it is a trade-off relationship between performance and efficiency. If we choose longer time interval window sizes, we can obtain better or worse results, due to adding noise in the time samples. The attacker must choose appropriate time intervals properly for successful analysis. Nevertheless, like the results in the previous section, TFA and TFAwPS have a mutual complementary performance in view of found key bytes (See Table A1 in the Appendix A).

### 4.5. Experimental Results on ASCAD Dataset

**Figure 11:**
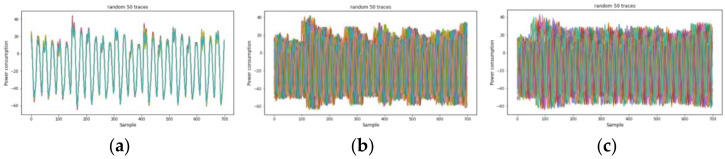
The power consumption traces for (**a**) ASCAD-sync, (**b**) ASCAD-desync50, and (**c**) ASCAD-desync100.

ASCAD is a well-known public dataset in the SCA domain for the reproducible study of profiling SCA, and it includes a set of electromagnetic (EM) traces obtained by measuring the emissions using an 8-bit MCU (ATMEGA8515), where a first-order masked AES operates [43]. The target of leakage is the 3rd SubByte in the 1st round of AES. ASCAD has three kinds of trace cases: ASCAD-sync, ASCAD-desync50, and ASCAD-desync100, as shown in Figure 11. The original traces are well aligned and are used as an ASCAD-sync case.

ASCAD-desync50 and ASCAD-desync100 are made by simulating uniform random shifting within 50 and 100, respectively, from the original synchronized traces. ASCAD includes 50,000 and 10,000 traces in each case for the profiling and attack phases, respectively, since ASCAD is a dataset for benchmarking profiling attacks. We used all 60,000 traces simultaneously, including profiling traces and attack traces, because we only investigated TFA and TFA with zero-mean preprocessing.

**Figure 12:**
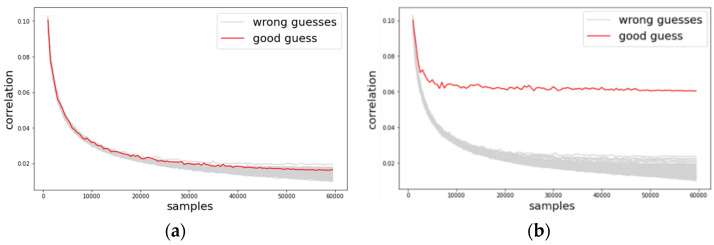
The results of the correlation analysis against ASCAD-sync (**a**) TFA and (**b**) TFAwPS.

**Figure 13:**
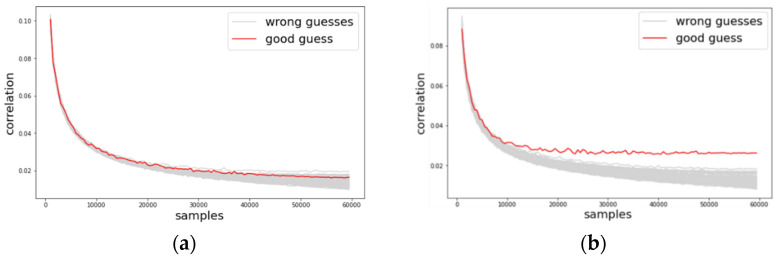
The results of the correlation analysis against ASCAD-desync50 (**a**) TFA and (**b**) TFAwPS.

**Figure 14:**
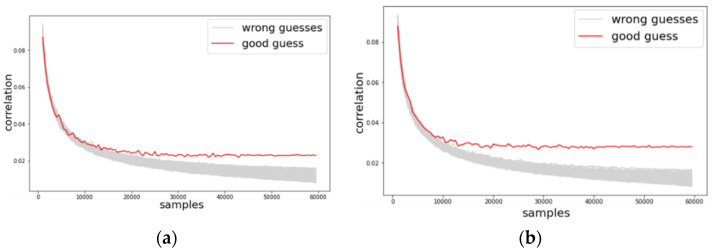
The results of the correlation analysis against ASCAD-desync100 (**a**) TFA and (**b**) TFAwPS.

**Figure 15:**
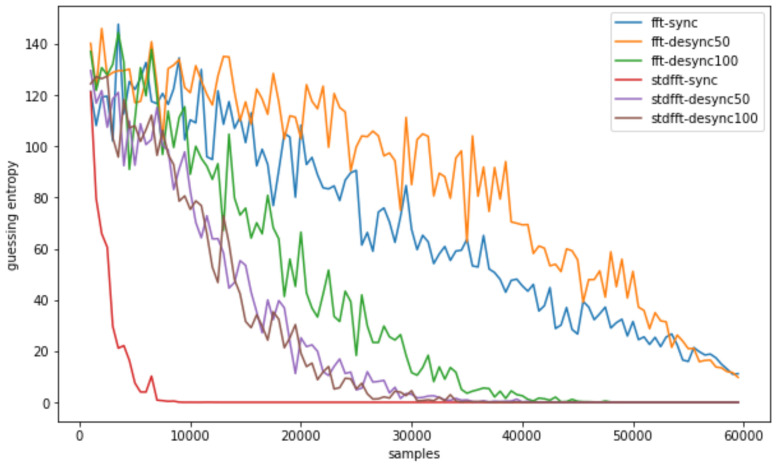
All guessing entropy results for TFA and TFAwPS against all cases of ASCAD.

Just as in the previous section, we conducted the same preprocessing and analysis methodologies on these datasets, employing the basic CPA, TFA, and TFAwPS against the ASCAD datasets. We present the results against each dataset according to varying the number of traces in Figure 12, Figure 13 and Figure 14. Note that TFA fails to find the key byte in all cases except for ASCAD-desync100. We cannot hypothesize why TFA can find the key against ASCAD-desync100. However, all results show that TFAwPS always succeeds in finding the key byte, and that TFAwPS outperforms TFA. Figure 15 illustrates the average guessing entropy on all ASCAD datasets per the number of traces. From these results, we again easily confirm that TFAwPS outperforms TFA for all ASCAD datasets. Therefore, all of the results on ASCAD datasets clearly support our claim.

## 5. Conclusions

We explored Belgarric et al.’s TFA. While their TFA can be used for SO-CPA with roughly chosen time intervals of leakages, and it has the potential to scale for higher-order attacks, it still performs poorly in some datasets. Because our mathematical analysis of the TFA shows that zero-mean preprocessing acts as a more suitable leakage term in situations when the constant noise is not zero or an equivalent leakage term otherwise, we claim that zero-mean preprocessing can enhance TFA performance. We conducted experiments and provided results supporting this claim. In the case of synchronized datasets, the TFA with zero-mean preprocessing achieves a better result than the TFA without preprocessing. In addition, in the case of desynchronized datasets, the TFA with zero-mean preprocessing has a mutual complementary result with the TFA without preprocessing. Such complementary results might be caused by a non-zero constant noise situation. However, it is difficult to determine whether the noise constant is zero or not in desynchronization situations. Therefore, based on both mathematical analysis and our experimental results, we conclude with a recommendation to use both the TFA with zero-mean preprocessing and without preprocessing for datasets in which masking and desynchronization countermeasures, such as random delay, are applied concurrently.

## Figures and Tables

**Table 1:** The comparison of results for TFA and TFAwPS against AES-M-sync.

	TFA	TFAwPS
Average max peak of absolute correlation coefficients	0.036785	0.042720
Average confidence (1st max peak/2nd max peak)	1.591291	1.840527
The number of found key bytes	16	16

**Table 2:** The comparison of the results for TFA and TFAwPS against AES-M-desync50.

	TFA	TFAwPS
Average max peak of absolute correlation coefficients	0.028241	0.027701
Average confidence (1st max peak/2nd max peak)	1.234980	1.200778
The number of found key bytes	12	13

**Table 3:** The comparison of results for TFA and TFAwPS against AES-M-desync100.

	TFA	TFAwPS
Average max peak of absolute correlation coefficients	0.024567	0.025794
Average confidence (1st max peak/2nd max peak)	1.083455	1.123166
The number of found key bytes	4	4

## Data Availability

Not applicable.

## Appendix A

We herein provide the supplementary results against some datasets for showing that zero-mean preprocessing enhances the performance of time-frequency analysis.

**Table A1:** The results of FO-CPA without preprocessing, TFA, and TFA with zero-mean preprocessing against AES-M and AES-RD. O/X represents either success or failure for key guesses.

AES-M-sync																
Byte	1	2	3	4	5	6	7	8	9	10	11	12	13	14	15	16
No preprocessing	X	X	X	X	X	X	X	X	X	X	X	X	X	X	X	X
TFA	O	O	O	O	O	O	O	O	O	O	O	O	O	O	O	O
TFAwPS	O	O	O	O	O	O	O	O	O	O	O	O	O	O	O	O
TFAwPN	O	O	O	O	O	O	O	O	O	O	O	O	O	O	O	O
AES-M-desync30																
Byte	1	2	3	4	5	6	7	8	9	10	11	12	13	14	15	16
No preprocessing	X	X	X	X	X	X	X	X	X	X	X	X	X	X	X	X
TFA	O	O	O	O	O	O	O	O	X	O	O	O	O	O	O	O
TFAwPS	O	O	O	O	O	O	O	O	O	O	O	O	O	O	O	O
TFAwPN	O	X	X	X	X	X	X	X	X	X	X	X	O	X	X	X
AES-M-desync50																
Byte	1	2	3	4	5	6	7	8	9	10	11	12	13	14	15	16
No preprocessing	X	X	X	X	X	X	X	X	X	X	X	X	X	X	X	X
TFA	X	X	X	O	O	O	O	O	X	O	O	O	O	O	O	O
TFAwPS	O	O	O	O	X	O	X	O	X	O	O	O	O	O	O	O
TFAwPN	O	X	X	O	O	X	X	X	O	X	X	X	X	X	X	X
AES-M-desync100																
Byte	1	2	3	4	5	6	7	8	9	10	11	12	13	14	15	16
No preprocessing	X	X	X	X	X	X	X	X	X	X	X	X	X	X	X	X
TFA	X	X	X	X	O	X	X	X	X	X	O	O	O	X	X	X
TFAwPS	X	X	X	O	X	X	X	X	X	X	O	O	O	X	X	X
TFAwPN	O	X	O	X	X	X	X	O	X	X	X	O	X	X	X	X
AES-RD ^1^																
Byte	1	2	3	4	5	6	7	8	9	10	11	12	13	14	15	16
No preprocessing	X	X	X	X	X	O	X	X	X	X	X	X	X	X	X	X
TFA	O	X	X	X	O	O	X	O	O	O	X	X	O	O	X	O
TFAwPS	O	X	X	O	O	O	O	O	O	O	X	O	O	O	X	O
TFAwPN	O	O	X	O	O	O	X	O	O	O	X	X	O	O	X	X

^1^ AES-RD dataset https://github.com/ikizhvatov/randomdelays-traces (accessed on 8 November 2021).

**Table A2:** The results of FO-CPA without preprocessing, TFA, and TFA with zero-mean preprocessing against ASCAD. O/X represents either success or failure for key guesses.

	ASCAD-sync	ASCAD-desync50	ASCAD-desync100
No preprocessing	X	X	X
TFA	X	X	O
TFAwPS	O	O	O
TFAwPN	O	X	X

## References

[1] Kocher P.C., Koblitz N. (1996). Timing Attacks on Implementations of Diffie-Hellman, RSA, DSS, and Other Systems. Advances in Cryptology—CRYPTO ’96, Proceedings of the 16th Annual International Cryptology Conference, Santa Barbara, CA, USA, 18–22 August 1996.

[2] Kocher P.C., Jaffe J., Jun B., Wiener M.J. (1999). Differential Power Analysis. Advances in Cryptology—CRYPTO ’99, Proceedings of the 19th Annual International Cryptology Conference, Santa Barbara, CA, USA, 15–19 August 1999.

[3] Gandolfi K., Mourtel C., Olivier F., Koç Ç.K., Naccache D., Paar C. (2001). Electromagnetic Analysis: Concrete Results. Cryptographic Hardware and Embedded Systems—CHES 2001, Proceedings of the Third International Workshop, Paris, France, 14–16 May 2001.

[4] Quisquater J.-J., Samyde D., Attali I., Jensen T.P. (2001). ElectroMagnetic Analysis (EMA): Measures and Counter-Measures for Smart Cards. Proceedings of the International Conference on Research in Smart Cards: Smart Card Programming and Security, E-Smart 2001, Cannes, France, 19–21 September 2001.

[5] Genkin D., Shamir A., Tromer E., Garay J.A., Gennaro R. (2014). RSA Key Extraction via Low-Bandwidth Acoustic Cryptanalysis. Advances in Cryptology—CRYPTO 2014, Proceedings of the 34th Annual Cryptology Conference, Santa Barbara, CA, USA, 17–21 August 2014.

[6] Hutter M., Schmidt J.-M., Francillon A., Rohatgi P. (2013). The Temperature Side Channel and Heating Fault Attacks. Smart Card Research and Advanced Applications, Proceedings of the 12th International Conference, CARDIS 2013, Berlin, Germany, 27–29 November 2013.

[7] Shepherd C., Markantonakis K., van Heijningen N., Aboulkassimi D., Gaine C., Heckmann T., Naccache D. (2021). Physical Fault Injection and Side-Channel Attacks on Mobile Devices: A Comprehensive Analysis. Comput. Secur..

[8] Mangard S., Oswald E., Popp T. (2007). Power Analysis Attacks—Revealing the Secrets of Smart Cards.

[9] Randolph M., Diehl W. (2020). Power Side-Channel Attack Analysis: A Review of 20 Years of Study for the Layman. Cryptography.

[10] Chari S., Rao J.R., Rohatgi P., Kaliski B.S., Koç K., Paar C. (2003). Template Attacks. Cryptographic Hardware and Embedded Systems—CHES 2002, Proceedings of the 4th International Workshop, Redwood Shores, CA, USA, 13–15 August 2002.

[11] Batina L., Djukanovic M., Heuser A., Picek S., Avoine G., Hernandez-Castro J. (2021). It Started with Templates: The Future of Profiling in Side-Channel Analysis. Security of Ubiquitous Computing Systems: Selected Topics.

[12] Maghrebi H., Portigliatti T., Prouff E., Carlet C., Hasan M.A., Saraswat V. (2016). Breaking Cryptographic Implementations Using Deep Learning Techniques. Security, Privacy, and Applied Cryptography Engineering, Proceedings of the 6th International Conference, SPACE 2016, Hyderabad, India, 14–18 December 2016.

[13] Cagli E., Dumas C., Prouff E., Fischer W., Homma N. (2017). Convolutional Neural Networks with Data Augmentation Against Jitter-Based Countermeasures—Profiling Attacks without Pre-Processing. Cryptographic Hardware and Embedded Systems—CHES 2017, Proceedings of the 19th International Conference, Taipei, Taiwan, 25–28 September 2017.

[14] Carbone M., Conin V., Cornelie M.-A., Dassance F., Dufresne G., Dumas C., Prouff E., Venelli A. (2019). Deep Learning to Evaluate Secure RSA Implementations. IACR Trans. Cryptogr. Hardw. Embed. Syst..

[15] Brier E., Clavier C., Olivier F., Joye M., Quisquater J.-J. (2004). Correlation Power Analysis with a Leakage Model. Cryptographic Hardware and Embedded Systems—CHES 2004, Proceedings of the 6th International Workshop Cambridge, MA, USA, 11–13 August 2004.

[16] Le T.-H., Clédière J., Canovas C., Robisson B., Servière C., Lacoume J.-L., Goubin L., Matsui M. (2006). A Proposition for Correlation Power Analysis Enhancement. Cryptographic Hardware and Embedded Systems—CHES 2006, Proceedings of the 8th International Workshop, Yokohama, Japan, 10–13 October 2006.

[17] Chari S., Jutla C.S., Rao J.R., Rohatgi P., Wiener M.J. (1999). Towards Sound Approaches to Counteract Power-Analysis Attacks. Advances in Cryptology—CRYPTO ’99, Proceedings of the 19th Annual International Cryptology Conference, Santa Barbara, CA, USA, 15–19 August 1999.

[18] Herbst C., Oswald E., Mangard S., Zhou J., Yung M., Bao F. (2006). An AES Smart Card Implementation Resistant to Power Analysis Attacks. Applied Cryptography and Network Security, Proceedings of the 4th International Conference, ACNS 2006, Singapore, 6–9 June 2006.

[19] Coron J.-S., Goubin L., Koç Ç.K., Paar C. (2000). On Boolean and Arithmetic Masking against Differential Power Analysis. Cryptographic Hardware and Embedded Systems—CHES 2000, Proceedings of the Second International Workshop, Worcester, MA, USA, 17–18 August 2000.

[20] Goubin L., Patarin J. (1999). DES and Differential Power Analysis the “Duplication” Method. Proceedings of the CHES 1999: Cryptographic Hardware and Embedded Systems, Worcester, MA, USA, 12–13 August 1999.

[21] Fumaroli G., Martinelli A., Prouff E., Rivain M., Biryukov A., Gong G., Stinson D.R. (2010). Affine Masking against Higher-Order Side Channel Analysis. Proceedings of the Selected Areas in Cryptography—Proceedings of the 17th International Workshop, SAC 2010, Waterloo, ON, Canada, 12–13 August 2010.

[22] Tiri K., Akmal M., Verbauwhede I. A Dynamic and Differential CMOS Logic with Signal Independent Power Consumption to Withstand Differential Power Analysis on Smart Cards. Proceedings of the 28th European Solid-State Circuits Conference.

[23] Popp T., Mangard S., Rao J.R., Sunar B. (2005). Masked Dual-Rail Pre-Charge Logic: DPA-Resistance without Routing Constraints. Cryptographic Hardware and Embedded Systems—CHES 2005, Proceedings of the 7th International Workshop, Edinburgh, UK, 29 August—1 September 2005.

[24] Chen C., Eisenbarth T., Shahverdi A., Ye X., Joye M., Moradi A. (2014). Balanced Encoding to Mitigate Power Analysis: A Case Study. Smart Card Research and Advanced Applications, Proceedings of the 13th International Conference, CARDIS 2014, Paris, France, 5–7 November 2014.

[25] Maghrebi H., Servant V., Bringer J., Peyrin T. (2016). There Is Wisdom in Harnessing the Strengths of Your Enemy: Customized Encoding to Thwart Side-Channel Attacks. Fast Software Encryption, Proceedings of the 23rd International Conference, FSE 2016, Bochum, Germany, 20–23 March 2016.

[26] Coron J.-S., Kizhvatov I., Clavier C., Gaj K. (2009). An Efficient Method for Random Delay Generation in Embedded Software. Cryptographic Hardware and Embedded Systems—CHES 2009, Proceedings of the 11th International Workshop, Lausanne, Switzerland, 6–9 September 2009.

[27] Coron J.S., Kizhvatov I. (2010). Analysis and Improvement of the Random Delay Countermeasure of CHES 2009. Proceedings of the 12th International Conference on Cryptographic Hardware and Embedded Systems, Santa Barbara, CA, USA, 17–20 August 2010.

[28] Veyrat-Charvillon N., Medwed M., Kerckhof S., Standaert F.-X., Wang X., Sako K. (2012). Shuffling against Side-Channel Attacks: A Comprehensive Study with Cautionary Note. Advances in Cryptology—ASIACRYPT 2012, Proceedings of the 18th International Conference on the Theory and Application of Cryptology and Information Security, Beijing, China, 2–6 December 2012.

[29] Woudenberg J.G.J.V., Witteman M.F., Bakker B. (2011). Improving Differential Power Analysis by Elastic Alignment. Proceedings of the Topics in Cryptology—CT-RSA 2011—The Cryptographers’ Track at the RSA Conference 2011, San Francisco, CA, USA, 14–18 February 2011.

[30] Muijrers R.A., Woudenberg J.G.J.V., Batina L. (2011). RAM: Rapid Alignment Method. Proceedings of the CARDIS 2011: Smart Card Research and Advanced Applications, Leuven, Belgium, 14–16 September 2011.

[31] Clavier C., Coron J.-S., Dabbous N., Koç Ç.K., Paar C. (2000). Differential Power Analysis in the Presence of Hardware Countermeasures. Cryptographic Hardware and Embedded Systems—CHES 2000, Proceedings of the Second International Workshop, Worcester, MA, USA, 17–18 August 2000.

[32] Le T.-H., Clédière J., Servière C., Lacoume J.-L. (2007). Noise Reduction in Side Channel Attack Using Fourth-Order Cumulant. IEEE Trans. Inf. Forensics Secur..

[33] Nagashima S., Homma N., Imai Y., Aoki T., Satoh A. (2007). DPA Using Phase-Based Waveform Matching against Random-Delay Countermeasure. Proceedings of the International Symposium on Circuits and Systems (ISCAS 2007).

[34] Durvaux F., Renauld M., Standaert F.-X., tot Oldenzeel L.v.O., Veyrat-Charvillon N., Mangard S. (2012). Efficient Removal of Random Delays from Embedded Software Implementations Using Hidden Markov Models. Smart Card Research and Advanced Applications, Proceedings of the 11th International Conference, CARDIS 2012, Graz, Austria, 28–30 November 2012.

[35] Pozo S.M.D., Standaert F.-X., Güneysu T., Handschuh H. (2015). Blind Source Separation from Single Measurements Using Singular Spectrum Analysis. Cryptographic Hardware and Embedded Systems—CHES 2015, Proceedings of the 17th International Workshop, Saint-Malo, France, 13–16 September 2015.

[36] Batina L., Hogenboom J., van Woudenberg J.G.J., Dunkelman O. (2012). Getting More from PCA: First Results of Using Principal Component Analysis for Extensive Power Analysis. Proceedings of the Topics in Cryptology—CT-RSA 2012—The Cryptographers’ Track at the RSA Conference 2012, San Francisco, CA, USA, 27 February–2 March 2012.

[37] Souissi Y., Guilley S., Danger J.-L., Mekki S., Duc G. (2010). Improvement of Power Analysis Attacks Using Kalman Filter. Proceedings of the Proceedings of the IEEE International Conference on Acoustics, Speech, and Signal Processing, ICASSP 2010.

[38] Charvet X., Pelletier H. (2005). Improving the DPA Attack Using Wavelet Transform. https://csrc.nist.rip/groups/STM/cmvp/documents/fips140-3/physec/papers/physecpaper14.pdf.

[39] Maghrebi H., Prouff E., Fan J., Gierlichs B. (2018). On the Use of Independent Component Analysis to Denoise Side-Channel Measurements. Constructive Side-Channel Analysis and Secure Design, Proceedings of the 9th International Workshop, COSADE 2018, Singapore, 23–24 April 2018.

[40] Debande N., Souissi Y., Elaabid M.A., Guilley S., Danger J.-L. (2012). Wavelet Transform Based Pre-Processing for Side Channel Analysis. Proceedings of the 45th Annual IEEE/ACM International Symposium on Microarchitecture, MICRO 2012, Workshops Proceedings.

[41] Kwon D., Kim H., Hong S. (2021). Non-Profiled Deep Learning-Based Side-Channel Preprocessing with Autoencoders. IEEE Access.

[42] Dworkin M.J., Barker E.B., Nechvatal J.R., Foti J., Bassham L.E., Roback E., Dray J.F. (2001). Advanced Encryption Standard (AES). https://nvlpubs.nist.gov/nistpubs/fips/nist.fips.197.pdf.

[43] Benadjila R., Prouff E., Strullu R., Cagli E., Dumas C. (2020). Deep Learning for Side-Channel Analysis and Introduction to ASCAD Database. J. Cryptogr. Eng..

[44] Messerges T.S., Koç Ç.K., Paar C. (2000). Using Second-Order Power Analysis to Attack DPA Resistant Software. Proceedings of the Cryptographic Hardware and Embedded Systems—CHES, Worcester, MA, USA, 17–18 August 2000.

[45] Prouff E., Rivain M., Bevan R. (2009). Statistical Analysis of Second Order Differential Power Analysis. IEEE Trans. Comput..

[46] Waddle J., Wagner D.A., Joye M., Quisquater J.-J. (2004). Towards Efficient Second-Order Power Analysis. Cryptographic Hardware and Embedded Systems—CHES 2004, Proceedings of the 6th International Workshop, Cambridge, MA, USA, 11–13 August 2004.

[47] Timon B. (2019). Non-Profiled Deep Learning-Based Side-Channel Attacks with Sensitivity Analysis. IACR Trans. Cryptogr. Hardw. Embed. Syst..

[48] Durvaux F., Standaert F.-X., Veyrat-Charvillon N., Mairy J.-B., Deville Y., Mangard S., Poschmann A.Y. (2015). Efficient Selection of Time Samples for Higher-Order DPA with Projection Pursuits. Constructive Side-Channel Analysis and Secure Design, Proceedings of the 6th International Workshop, COSADE 2015, Berlin, Germany, 13–14 April 2015.

[49] Belgarric P., Bhasin S., Bruneau N., Danger J.-L., Debande N., Guilley S., Heuser A., Najm Z., Rioul O., Francillon A., Rohatgi P. (2013). Time-Frequency Analysis for Second-Order Attacks. Smart Card Research and Advanced Applications, Proceedings of the 12th International Conference, CARDIS 2013, Berlin, Germany, 27–29 November 2013.

[50] Gebotys C.H., Ho S., Tiu C.C., Rao J.R., Sunar B. (2005). EM Analysis of Rijndael and ECC on a Wireless Java-Based PDA. Cryptographic Hardware and Embedded Systems—CHES 2005, Proceedings of the 7th International Workshop, Edinburgh, UK, 29 August—1 September 2005.

[51] Chen J., Ng J.-S., Chong K.-S., Lin Z., Gwee B.-H. (2021). A Novel Normalized Variance-Based Differential Power Analysis against Masking Countermeasures. IEEE Trans. Inf. Forensics Secur..

[52] NewAE Technology CW308T-STM32F. https://rtfm.newae.com/Targets/UFO%20Targets/CW308T-STM32F/.

[53] NewAE Technology CW1173 ChipWhisperer-Lite. https://rtfm.newae.com/Capture/ChipWhisperer-Lite/.

